# Nano Minerals Applications in Pollutants Removal: A New Open Special Issue in *Materials*

**DOI:** 10.3390/ma15196704

**Published:** 2022-09-27

**Authors:** Po-Hsiang Chang, Raj Mukhopadhyay

**Affiliations:** 1 College of Resources and Environment, Fujian Agriculture and Forestry University, Fuzhou 350002, China; 2 Division of Irrigation and Drainage Engineering, ICAR-Central Soil Salinity Resaerch Institute, Karnal 132001, India

‘Nano Minerals Applications in Pollutants Removal: A New Open Special Issue in *Materials*’, aims to publish high-quality research and review articles on the basic and applied science of clay mineralogy and make great contributions to the understanding of applied mineralogy in removing pollutants from aqueous and soil environments [1].

Inorganic (heavy metals and metalloids, nitrate, selenium, fluoride, phosphate, etc.) pollutants in water and soil environments have negative impacts on living organisms due to their toxicological properties and cause damage to humans (disrupt liver function, brain, skin, abdominal pain, vomiting, and cancer). Similarly, the potential risks of different organic pollutants (Polyaromatic hydrocarbons, persistent organic pollutants (POPs), plastic polymers, per- and polyfluoro alkyl substances (PFAS), pesticides, dyes, medicinal drugs, etc.) in the environment can induce bacterial resistance, disrupt the balance of the ecosystem, and affect the growth and development of plants. Once they enter the human body through the food chain, they can cause allergic reactions and, in severe cases, food poisoning. Similar to heavy metals and metalloids, some drugs, pesticides, PFAS, and POPs also have carcinogenic, teratogenic, mutagenic, or hormonal effects, which seriously interfere with various human physiological functions and threaten human health. Thus, removing different pollutants from the aqueous and soil system is very much important to reducing the bioavailability of toxic pollutant chemicals to humans in order to achieve the United Nation’s Sustainable Development Goals [2]. In water treatment technologies, many adsorbents, such as carbon-based adsorbents, nanomaterials, metal-organic frameworks, etc., have been used widely; however, they suffer from high synthesis costs, energy requirements, and a lack of handling protocols. Therefore, inexpensive, novel, and highly efficient natural/modified adsorbents are the need of the hour for tackling pollutants in the environment. The use of naturally available clay minerals (even nano-sized) has huge potential to remove bulk amounts of pollutants through adsorption due to their high specific surface area, cation exchange capacity, no harmful impacts on living organisms, practical use, and huge natural abundance. Moreover, their inorganic and organic low-cost modifications of these clay minerals also hold better potential for environmental cleanup technology. The applications of various natural and modified clay minerals (bentonite, montmorillonite, palygorskite, kaolinite, illite, etc.) for soil and water remediation improve the understanding and knowledge of the colloid–pollutant interface, mechanisms, and environmental factors of adsorption, and thermodynamic relationship.

The research interest of this section on Nano Minerals Applications in Pollutants Removal includes, but is not limited to, the following: Natural clay minerals, organic and inorganic modifications including modifications with biochar, nanomaterials for removal of a wide range of water pollutants (both inorganic and organic) including contaminants of emerging concern from the environment.

## Data Availability

The data that support the findings of this study are openly available in the Chapter 7 of one Book [Modified Clay and Zeolite Nanocomposite Materials: Environmental and Pharmaceutical Applications], reference number [1].
